# Pregnancy‐Associated Spontaneous Coronary Artery Dissection in the Third Trimester Requiring Multistent Percutaneous Coronary Intervention: A Case Report

**DOI:** 10.1155/cric/5560692

**Published:** 2026-09-24

**Authors:** Steven Liu, Austin Layton, Hollace Chastain

**Affiliations:** ^1^ Department of Internal Medicine, Parkview Health, Fort Wayne, Indiana, USA; ^2^ Marian University Wood College of Osteopathic Medicine, Indianapolis, Indiana, USA; ^3^ Department of Cardiology, Parkview Health, Fort Wayne, Indiana, USA

## Abstract

**Background:**

Pregnancy‐associated spontaneous coronary artery dissection (P‐SCAD) is an uncommon cause of acute coronary syndrome (ACS) in young women. Although SCAD accounts for less than 1% of ACS cases, it causes up to 40%–50% of myocardial infarctions during pregnancy and the postpartum period. Third‐trimester cases requiring complex percutaneous coronary intervention (PCI) are particularly rare.

**Case Presentation:**

A 33‐year‐old gravida 5, para 3 woman at 27 weeks′ gestation presented with sudden severe substernal chest pain. Electrocardiographic findings and elevated cardiac biomarkers confirmed an anterior ST‐segment elevation myocardial infarction (STEMI). Coronary angiography demonstrated spontaneous coronary artery dissection involving the mid‐to‐distal left anterior descending artery. Owing to ongoing ischemia and extensive dissection, she underwent intravascular ultrasound‐guided PCI with four overlapping drug‐eluting stents, restoring TIMI III flow. Following revascularization, she remained hospitalized for maternal and fetal monitoring because of concerns for preeclampsia and severe fetal growth restriction. Although expectant management was initially pursued, worsening fetal heart‐rate concerns necessitated cesarean delivery at 29 weeks′ gestation without maternal cardiac complications. Although left ventricular function initially improved with guideline‐directed medical therapy (GDMT), discontinuation of several medications because she felt symptomatically better contributed to progressive left ventricular dysfunction and NYHA class III heart failure, ultimately requiring cardiac rehabilitation and prophylactic implantable cardioverter‐defibrillator placement.

**Discussion:**

This case highlights the complexity of managing extensive third‐trimester P‐SCAD requiring multistent PCI while balancing maternal cardiovascular stabilization with ongoing obstetric complications. It further emphasizes the importance of longitudinal imaging, sustained GDMT, medication adherence, and coordinated multidisciplinary follow‐up after significant myocardial injury.

**Conclusion:**

Third‐trimester P‐SCAD requiring complex PCI is rare. Early recognition, individualized revascularization strategies, multidisciplinary obstetric and cardiovascular management, and close long‐term follow‐up are essential to optimize maternal and fetal outcomes while reducing the risk of adverse ventricular remodeling and heart failure progression.

## 1. Introduction

SCAD is a nonatherosclerotic cause of ACS that predominantly affects young women without traditional cardiovascular risk factors. It results from separation of the coronary arterial wall, leading to formation of an intramural hematoma or false lumen that can compromise coronary blood flow and result in myocardial infarction, ventricular dysfunction, arrhythmia, or sudden cardiac death [1, 2]. Although SCAD accounts for a small proportion of ACS presentations overall, it is disproportionately observed among young and peripartum women and represents an important cause of pregnancy‐associated myocardial infarction [1, 3–5].

P‐SCAD is thought to arise from a combination of hormonal, hemodynamic, and vascular adaptations during pregnancy. Elevated progesterone and estradiol levels may contribute to vascular fragility through alterations in collagen synthesis and elastic fiber integrity, whereas increased blood volume and cardiac output during late gestation increase arterial wall shear stress. Additional reported associations include multiparity, hypertensive disorders of pregnancy, connective tissue disorders, and significant physical or emotional stressors [2, 3, 6].

P‐SCAD most commonly occurs during the peripartum period or early postpartum, although mid‐to‐late gestation presentations have been described and present unique diagnostic and therapeutic challenges [3–5, 7–10]. Management requires individualized assessment balancing maternal cardiovascular stability with fetal safety, including decisions regarding coronary angiography, antiplatelet therapy, timing of delivery, and pregnancy‐specific limitations of guideline‐directed therapies [6, 11].

Unlike atherosclerotic ACS, conservative management is generally preferred in SCAD because of the risk of iatrogenic extension during PCI [1, 6]. However, revascularization may be required in patients with ongoing ischemia, extensive coronary involvement, or hemodynamic instability [1, 6]. PCI remains technically challenging because of fragile arterial walls, long dissected segments, and difficulty identifying the true lumen [1, 12]. Consequently, extensive multistent PCI during pregnancy remains exceedingly uncommon, with only isolated cases reported [13, 14].

We present a rare case of third‐trimester P‐SCAD presenting as anterior STEMI with extensive mid‐to‐distal LAD involvement requiring four overlapping drug‐eluting stents. This case highlights the challenges of invasive management during pregnancy and the importance of multidisciplinary decision‐making to optimize maternal cardiac recovery and fetal outcomes.

## 2. Case Summary

A 33‐year‐old gravida 5, para 3 woman at 27 weeks′ gestation with a history of tobacco use presented with sudden‐onset, severe substernal chest pain radiating to her left arm. She was hypertensive but remained hemodynamically stable. Electrocardiography demonstrated an anterior STEMI with corresponding elevation in cardiac biomarkers, prompting emergent transfer to the cardiac catheterization laboratory.

**Figure 1:**
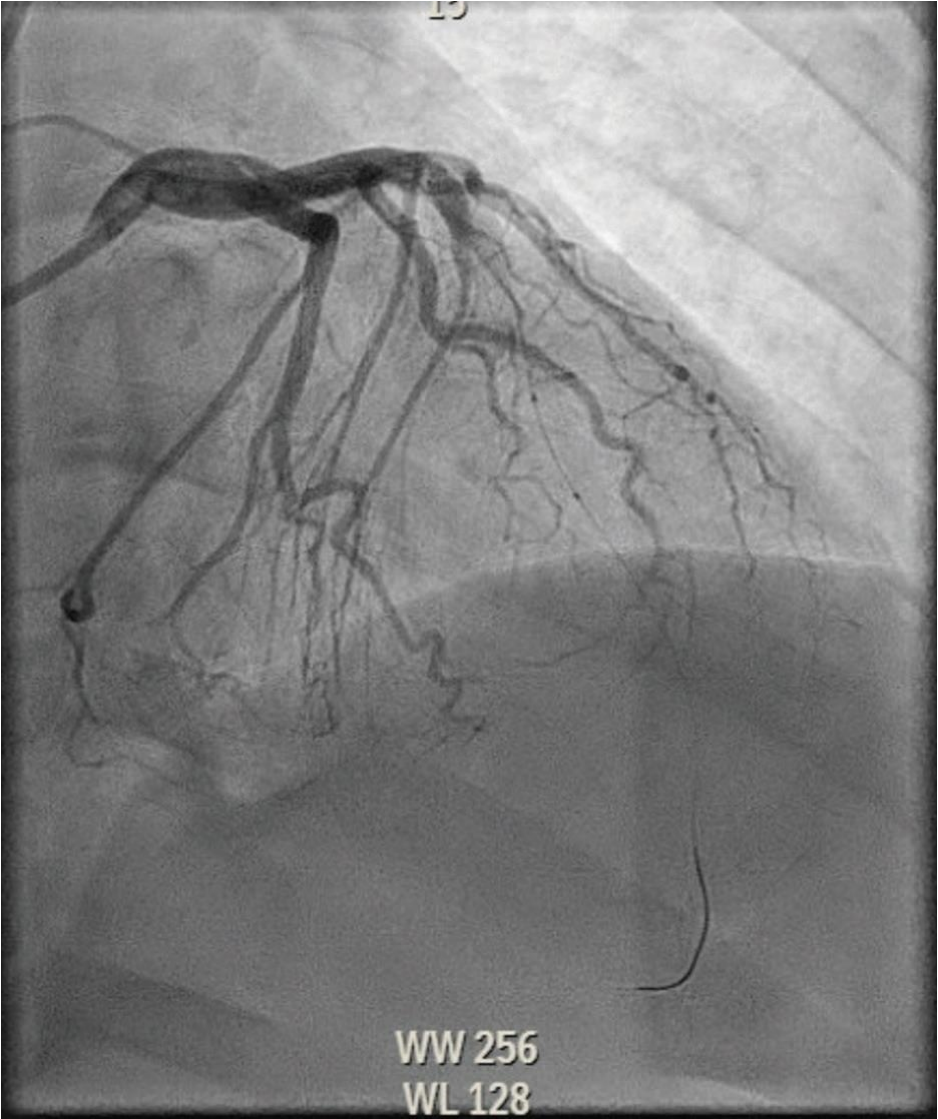
Initial coronary angiography showed total occlusion of mid to distal LAD secondary to spontaneous coronary artery dissection.

**Figure 2:**
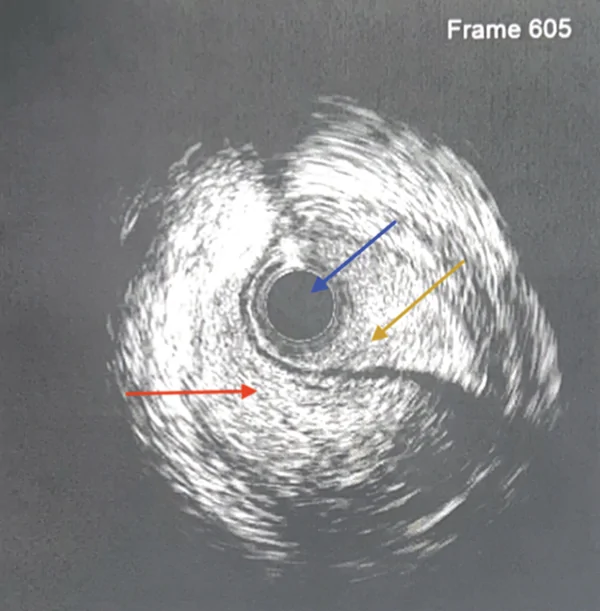
Prestenting intravascular ultrasound (IVUS) image of the left anterior descending artery. IVUS confirmed positioning of the imaging catheter within the distal true lumen and demonstrated an eccentric intramural hematoma with associated true‐lumen compression before stent deployment. The blue arrow indicates the IVUS catheter, the yellow arrow indicates the true lumen, and the red arrow indicates the intramural hematoma.

**Figure 3:**
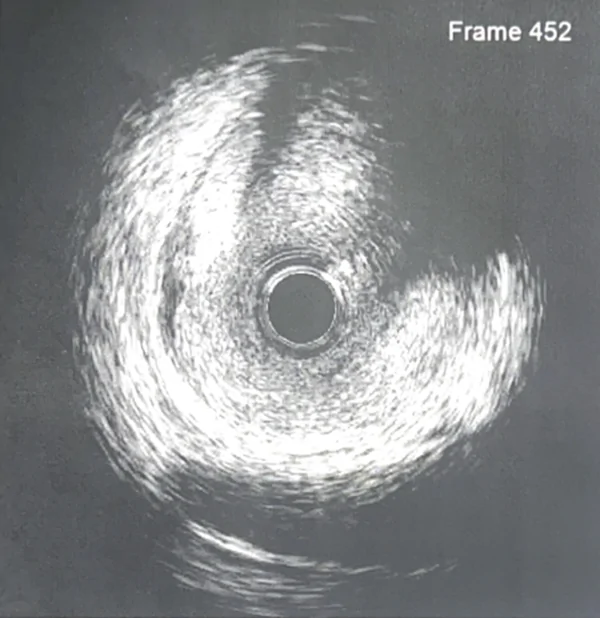
Poststenting intravascular ultrasound (IVUS) image of the left anterior descending artery. Following deployment of four overlapping drug‐eluting stents, IVUS demonstrates scaffolding and expansion of the true lumen within the treated segment.

**Figure 4:**
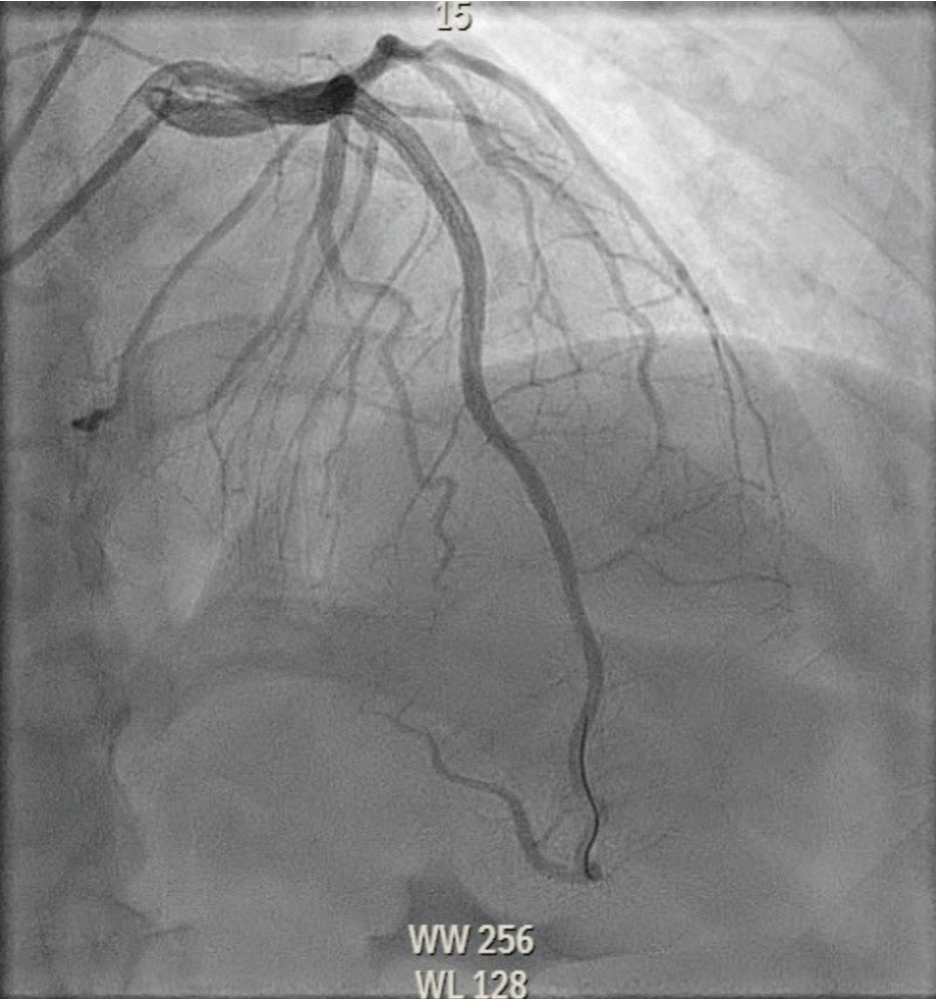
Successful IVUS guided intervention of the mid to apical LAD with four overlapping drug eluting stents.

Coronary angiography revealed SCAD involving the mid‐to‐distal LAD with complete loss of distal coronary flow (TIMI 0) due to true‐lumen compromise (Figure 1). Given the acute STEMI, ongoing myocardial ischemia, and absent distal perfusion, immediate revascularization was pursued rather than conservative management. Initial conventional balloon angioplasty failed to restore flow and appeared to propagate the dissection proximally. IVUS confirmed distal true‐lumen wire position and demonstrated an intramural hematoma causing true‐lumen compression (Figure 2). Because distal flow remained absent despite balloon angioplasty, the decision was made to intervene on the vessel to restore coronary perfusion. Three overlapping drug‐eluting stents were initially deployed (2.0 × 30 mm, 2.5 × 38 mm, and 2.0 × 15 mm Onyx Frontier stents), partially restoring distal flow; however, flow limitation persisted in association with proximal extension of the intramural hematoma. Cutting‐balloon angioplasty was attempted to decompress the hematoma but did not improve flow. Repeat IVUS was therefore used to identify a healthy proximal landing zone, followed by placement of a fourth overlapping drug‐eluting stent (3.5 × 38 mm Onyx Frontier) (Figure 3). The four stents spanned the mid‐to‐apical LAD and restored TIMI III flow with preservation of the diagonal and septal branches (Figure 4). The residual distal edge dissection appeared improved and was nonflow limiting; therefore, further intervention was deferred.

Given her pregnancy status, coronary angiography and PCI were performed with consideration of maternal and fetal safety. Radiation exposure was managed according to the ALARA (as low as reasonably achievable) principle, with fluoroscopic and angiographic projections selected to minimize direct radiation exposure to the maternal abdomen and pelvis whenever feasible. In this case, steep projection angles were specifically used, when necessary, to direct the primary radiation field away from the abdomen. Abdominal/pelvic lead shielding was not used following discussion between the interventional cardiologist and cath lab medical director. Total fluoroscopy time was 22.1 min, with a reported procedural radiation dose of 920.490 mGy and dose‐area product of 81.600 Gy·cm^2^. A total of 250 mL of iohexol contrast was administered. Fetal heart rate was 138 beats/min on admission and was documented at approximately 145–160 beats/min during and after the procedure, with fetal movement visualized. Given the ongoing anterior STEMI, complete distal LAD flow compromise, and risk of continued maternal myocardial injury, the anticipated maternal and fetal benefits of urgent revascularization were considered to outweigh the potential risks associated with procedural radiation and iodinated contrast exposure.

Postprocedure management included dual antiplatelet therapy (DAPT) with aspirin and clopidogrel, intravenous heparin because of significant apical dysfunction and concern for left ventricular thrombus formation, and carvedilol therapy. Angiotensin‐converting enzyme inhibitors and angiotensin receptor blockers were deferred during pregnancy because of fetal safety considerations. Given the contraindication to renin–angiotensin system inhibitors during pregnancy, hydralazine and nitrates were considered pregnancy–compatible alternatives for afterload reduction as part of HFrEF medical management. However, initiation was deferred because of low blood pressure and concern that nitrate‐associated headaches could complicate the assessment of neurologic symptoms in the setting of suspected preeclampsia. Following PCI, she remained hospitalized for multidisciplinary maternal and fetal monitoring because of concerns for preeclampsia and fetal growth restriction. Given maternal and fetal stability after coronary intervention, expectant inpatient management was initially pursued with the goal of prolonging pregnancy to approximately 34 weeks′ gestation to optimize fetal maturity while monitoring for maternal cardiac deterioration and worsening obstetric complications. However, worsening fetal heart rate concerns necessitated delivery. Additionally, because she remained on uninterrupted DAPT following recent drug‐eluting stent implantation, neuraxial anesthesia was contraindicated because of the risk of spinal epidural hematoma. After multidisciplinary discussion among maternal‐fetal medicine, cardiology, obstetrics, and anesthesiology, the risks of continued pregnancy were determined to outweigh the potential benefits of additional fetal maturation. Cesarean delivery was therefore performed at 29 weeks′ gestation under general anesthesia. Despite continuation of DAPT throughout the peripartum period, she experienced no significant perioperative bleeding or hemorrhagic complications. A male infant was delivered weighing 2 lb 1.4 oz with Apgar scores greater than 7 at 1 and 5 min. The neonate was admitted to the neonatal intensive care unit for management of extreme prematurity and respiratory distress syndrome, with a prolonged NICU course lasting approximately 4–5 months before discharge.

Serial echocardiographic evaluations demonstrated an initial LVEF of 35%–40% with distal septal and apical akinesis and possible apical thrombus. Follow‐up imaging showed improvement in LVEF to 47% with mild apical trabeculation and no definitive thrombus. Postpartum, GDMT was expanded and optimized as tolerated with initiation of metoprolol and benazepril, whereas DAPT with aspirin and clopidogrel and tobacco cessation counseling were continued at the time of discharge.

Approximately 4 months after delivery, the patient was rehospitalized with recurrent chest pain, subtle electrocardiographic changes, and mildly elevated troponin levels. During this admission, she reported discontinuing DAPT, metoprolol, and benazepril approximately 2 months earlier because she noted symptomatic improvement and perceived that continued medication therapy was no longer necessary. This represented patient‐driven medication nonadherence rather than medication intolerance, hypotension, or another medical contraindication. Notably, DAPT had been interrupted despite placement of four overlapping LAD drug‐eluting stents, representing a significant concern given the extent of prior coronary intervention. During this period of medication discontinuation, her ventricular function worsened, with subsequent echocardiography demonstrating a decline in LVEF to 28%, raising concern for progressive ischemic cardiomyopathy. Repeat coronary angiography was planned due to concern for recurrent ischemia; however, she left the hospital against medical advice prior to undergoing the procedure.

**Figure 5:**
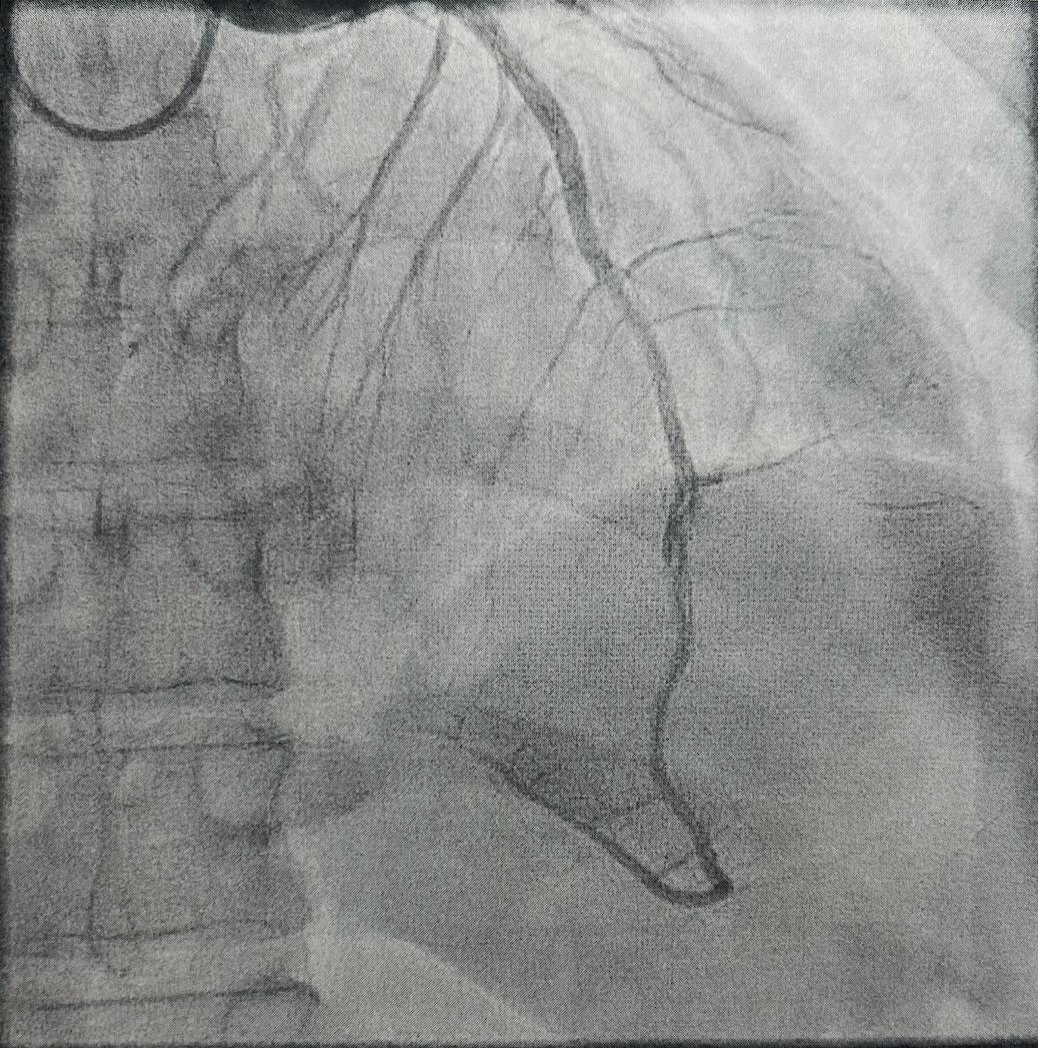
Diagnostic coronary angiography showed chronic residual distal LAD dissection, which is already covered by previous stenting, which was difficult to determine lesion severity, but with TIMI3 flow appears nonobstructive and estimated 40% stenosed.

At subsequent outpatient follow‐up, she continued to experience intermittent chest and back discomfort, palpitations, and exertional dyspnea with persistent ventricular dysfunction. Given persistent symptoms, reduced LVEF despite reinitiation of GDMT, and concern regarding her prior extensive LAD intervention, repeat coronary angiography was performed. The study demonstrated a chronic residual distal LAD dissection within the previously stented segment with preserved TIMI III flow and approximately 40% residual distal stenosis. As this lesion was considered nonobstructive and unlikely to account for her persistent cardiomyopathy, repeat PCI was deferred in favor of continued medical therapy and aggressive risk factor modification (Figure 5).

Given the extensive LAD territory infarction and initial severe ventricular dysfunction, she remained at risk for adverse ventricular remodeling despite successful revascularization. She subsequently developed NYHA class III symptoms with persistent severe left ventricular systolic dysfunction, with repeat echocardiography demonstrating LVEF values of 28%–29% despite reinitiation of GDMT. Further optimization of heart failure therapy was limited by persistent hypotension, which prevented additional titration of beta‐blocker therapy and benazepril and precluded initiation or escalation of other guideline‐directed therapies, including ACE inhibitor/ARB/ARNI, sodium–glucose cotransporter‐2 inhibitor, and mineralocorticoid receptor antagonist therapy. After completing cardiac rehabilitation and an appropriate recovery period following myocardial infarction and revascularization, she continued to have LVEF ≤ 35% despite maximally tolerated GDMT. She subsequently underwent uncomplicated dual‐chamber ICD placement for primary prevention of sudden cardiac death. A chronological overview of the patient′s clinical course, cardiac findings, management, and outcomes is provided in Table 1.

**Table 1:** Chronological timeline of clinical course, cardiac findings, and management.

Time	Clinical events	Cardiac findings	Management/outcome
Hospital Day 1: 27 weeks gestation	Sudden substernal chest pain radiating to left arm	Anterior STEMI; angiography showed mid‐distal LAD‐SCAD with TIMI 0 flow	Conventional balloon angioplasty → IVUS assessment → three overlapping DES implanted → cutting‐balloon angioplasty and repeat IVUS for persistent flow limitation and proximal intramural hematoma extension → fourth DES implanted, restoring TIMI III flow.
Hospital Days 2–13	Inpatient maternal‐fetal monitoring	Initial LVEF 35%–40%; distal septal/apical akinesis; possible LV thrombus	DAPT, IV heparin, carvedilol; expectant management for fetal maturity
Hospital Day 14: 29 weeks gestation	Worsening fetal heart rate concerns	Maternal cardiac status stable after PCI	Cesarean delivery under general anesthesia (DAPT precluded neuraxial anesthesia); infant admitted to NICU
Hospital discharge	Postpartum recovery	LVEF improved to 47%; no definite LV thrombus	Switched from carvedilol to metoprolol, initiated benazepril, continued aspirin/clopidogrel, tobacco cessation counseling
~2 months postpartum	Medication interruption	Patient asymptomatic	Self‐discontinued aspirin, clopidogrel, metoprolol, and benazepril after symptomatic improvement
~4 months postpartum	Readmission with chest pain	Mild troponin elevation; LVEF declined to 28%	Planned repeat angiography; patient left AMA before procedure
~8 months outpatient follow‐up	Persistent chest/back pain, dyspnea, palpitations	Residual distal LAD dissection with TIMI III flow; ~40% distal stenosis	Medical management; repeat PCI deferred
~12 months long‐term follow‐up	Persistent ischemic cardiomyopathy	LVEF 28%–29%; NYHA class III despite maximally tolerated GDMT	Completed cardiac rehabilitation; dual‐chamber ICD implanted for primary prevention

## 3. Discussion

P‐SCAD is rare but clinically significant in young women. Most cases occur during the peripartum or early postpartum period, making third‐trimester presentations uncommon and diagnostically challenging [1, 3, 4, 11]. Acute coronary syndrome should remain an important diagnostic consideration in pregnant patients presenting with chest pain, even in the absence of traditional cardiovascular risk factors, as delayed recognition may result in irreversible myocardial injury and adverse maternal outcomes [1, 3, 4].

The extent and location of the dissection contributed to the complexity of this case. Although the LAD is the most frequently affected coronary artery in SCAD, dissections typically involve proximal segments [1, 15]. Mid‐to‐distal LAD involvement is less common and presents technical challenges for PCI due to smaller distal caliber, limited flow reserve, and difficulty achieving complete lesion coverage [2, 6]. In this patient, four overlapping drug‐eluting stents were required to restore TIMI III flow, reflecting the extensive dissection burden and procedural complexity [13, 14]. Unlike atherosclerotic PCI, intervention in SCAD requires careful consideration of intramural hematoma extent, risk of propagation, and the need to adequately restore flow while minimizing unnecessary vessel manipulation [1, 2, 6].

IVUS provided important diagnostic and procedural guidance in this case. Intracoronary imaging can help confirm the presence and extent of intramural hematoma, distinguish true‐lumen compression from other causes of coronary obstruction, and guide appropriate stent sizing and deployment [1, 2, 6]. However, IVUS use in SCAD must be balanced against the potential risk of extending the dissection in fragile coronary vessels [1, 6]. In this patient, IVUS‐assisted PCI facilitated treatment of extensive LAD involvement while optimizing stent placement and restoration of coronary flow.

Distal anterior and apical dyskinesis with possible mural thrombus reflected severe ischemic injury in this patient. Although regional wall motion abnormalities are common in LAD‐SCAD, extensive dyskinesis and mural thrombus are infrequently reported [16]. Restoration of coronary blood flow does not necessarily result in immediate recovery of ventricular function, particularly when significant myocardial injury has already occurred [1, 12]. Given the extensive LAD territory infarction, the initial ischemic insult carried substantial risk for adverse ventricular remodeling despite successful revascularization. Therefore, recovery after P‐SCAD should not be assessed solely by restoration of coronary flow but should include serial evaluation of ventricular function and early initiation and optimization of GDMT when systolic dysfunction is present [17].

Reported cases of third‐trimester LAD‐SCAD have generally been managed conservatively or with limited PCI. Paxton‐Hall et al. (2021), Vongbunyong et al. (2023), and Tsianakas et al. (2025) described third‐trimester SCAD involving the LAD without requiring multistent PCI [7, 9, 10]. Wingerter et al. (2021) reported a patient requiring emergent intervention because of cardiogenic shock but without extensive multistent stenting [8]. This case represents a rare example of extensive mid‐to‐distal LAD dissection requiring four drug‐eluting stents with associated severe myocardial injury and persistent ventricular dysfunction.

Although conservative management is preferred in many SCAD cases because of the potential for spontaneous healing and procedural risks associated with intervention, PCI remains appropriate in selected high‐risk presentations. Indications for revascularization include persistent or recurrent ischemia despite medical therapy, ongoing ST‐segment elevation, hemodynamic instability, ventricular arrhythmias, proximal or left main involvement, or failure to achieve adequate coronary flow [1, 2, 6, 12]. In this patient, emergent PCI was pursued because of anterior STEMI, complete distal LAD flow compromise, and ongoing myocardial jeopardy. Importantly, the initial attempt at stent‐sparing therapy failed to restore adequate perfusion, necessitating bailout PCI. This case highlights that P‐SCAD management requires individualized decision‐making rather than a universal conservative or invasive approach.

PCI during pregnancy requires careful balancing of maternal and fetal risks, including radiation exposure, contrast administration, and obstetric bleeding considerations [1, 11, 15]. Pregnancy should not delay indicated invasive evaluation in patients with STEMI, as the risk of untreated maternal ischemia may outweigh procedural risks when appropriate radiation‐minimization strategies are used [1, 11]. In this case, radiation exposure was minimized through limited fluoroscopic imaging and cineangiographic acquisitions, consistent with the ALARA principle. In this case, radiation exposure was managed in accordance with the ALARA principle. Given the ongoing anterior STEMI, complete distal LAD flow compromise, and risk of continued maternal myocardial injury, the benefits of urgent revascularization were considered to outweigh the potential maternal and fetal risks associated with radiation and iodinated contrast exposure.

Post‐PCI management in pregnancy requires additional consideration because of the competing risks of stent thrombosis and peripartum bleeding. DAPT is recommended after drug‐eluting stent placement; however, therapy must be individualized based on gestational age, procedural complexity, and delivery planning [1, 11, 15]. In this patient, the need for uninterrupted DAPT following extensive LAD stenting created significant peripartum management challenges. Cesarean delivery was ultimately performed because of worsening fetal heart rate concerns and the inability to safely discontinue clopidogrel to permit neuraxial anesthesia. Management required multidisciplinary coordination among cardiology, maternal‐fetal medicine, obstetrics, and anesthesiology to balance maternal ischemic risk, stent thrombosis prevention, fetal status, and bleeding concerns [11, 18]. This case emphasizes the importance of early multidisciplinary planning when PCI occurs late in pregnancy.

Long‐term follow‐up is essential following P‐SCAD, particularly in patients with extensive myocardial injury. SCAD is a dynamic disease process in which healing, remodeling, and residual coronary abnormalities may occur over time; therefore, follow‐up angiography should be interpreted in conjunction with clinical status and ventricular recovery rather than as the sole determinant of outcome [1, 12, 16]. In this patient, follow‐up angiography demonstrated preserved distal LAD flow and a nonflow‐limiting residual distal dissection, allowing continued medical management rather than repeat intervention. However, despite successful revascularization, significant LV dysfunction persisted, highlighting the importance of longitudinal assessment beyond angiographic improvement.

Persistent LV dysfunction following SCAD‐related myocardial infarction requires ongoing management with GDMT optimization, reassessment of ventricular recovery, and consideration of ICD implantation when appropriate [17]. In this patient, repeat echocardiography demonstrated persistent LVEF of approximately 28%–29% despite maximally tolerated GDMT after an appropriate recovery period, prompting evaluation for primary prevention ICD placement. This highlights that young patients with SCAD may experience long‐term complications requiring heart failure and electrophysiology follow‐up despite successful acute intervention [17].

Medication adherence represents an additional challenge in the postpartum period. Symptomatic improvement does not necessarily indicate complete myocardial recovery or resolution of thrombotic risk. In this case, patient‐driven discontinuation of antiplatelet therapy and GDMT after clinical improvement contributed to interruption of secondary prevention strategies. This underscores the importance of counseling patients regarding the rationale for continued therapy and the need for long‐term cardiovascular follow‐up.

Fewer than 10 third‐trimester P‐SCAD cases requiring PCI have been reported, with only a subset involving multistent LAD interventions [7–10, 13, 14, 19]. This case adds to the limited literature by demonstrating complex mid‐to‐distal LAD dissection requiring four overlapping drug‐eluting stents, severe myocardial injury with persistent LV dysfunction, and the importance of individualized revascularization decisions, intracoronary imaging when appropriate, coordinated peripartum management, and longitudinal cardiovascular follow‐up in complex P‐SCAD.

## 4. Conclusion

P‐SCAD is a rare but potentially life‐threatening cause of acute coronary syndrome in young women [1, 15]. This case is distinguished by its third‐trimester presentation, extensive mid‐to‐distal LAD involvement requiring four overlapping drug‐eluting stents, and severe distal anterior/apical dyskinesis with possible mural thrombus formation [2, 6]. Although conservative management remains appropriate for many patients with SCAD, this case highlights the importance of individualized decision‐making when persistent ischemia, extensive coronary involvement, or compromised myocardial perfusion necessitates invasive intervention. The use of intracoronary imaging, careful PCI planning, and multidisciplinary coordination were essential in achieving successful maternal and fetal outcomes [1, 11, 13].

Postpartum management requires continued surveillance beyond the acute event, including medication adherence, optimization of guideline‐directed medical therapy, serial assessment of left ventricular recovery, and risk‐factor modification [2, 12, 15]. Despite successful revascularization, extensive myocardial injury may result in persistent ventricular dysfunction requiring long‐term heart failure management and risk stratification for ICD therapy when indicated [17]. Given the rarity of complex multistent PCI in late‐pregnancy SCAD, this report provides insight into invasive treatment strategies, peripartum antiplatelet management, follow‐up assessment of coronary healing, and the importance of longitudinal cardiovascular care in high‐risk patients. Ultimately, prompt recognition, individualized intervention, and collaborative multidisciplinary management are essential to optimize outcomes in patients with complex P‐SCAD [1, 11–13].

## Funding

No funding was received for this manuscript.

## Consent

Written informed consent was obtained from the patient for the publication of this case report and any accompanying images.

## Conflicts of Interest

The authors declare no conflicts of interest.

## Data Availability

Data sharing is not applicable to this article as no datasets were generated or analyzed during the current study.
